# The Role of the Metabolism of Zinc and Manganese Ions in Human Cancerogenesis

**DOI:** 10.3390/biomedicines10051072

**Published:** 2022-05-05

**Authors:** Julian Markovich Rozenberg, Margarita Kamynina, Maksim Sorokin, Marianna Zolotovskaia, Elena Koroleva, Kristina Kremenchutckaya, Alexander Gudkov, Anton Buzdin, Nicolas Borisov

**Affiliations:** 1 Moscow Institute of Physics and Technology, National Research University, 141700 Moscow, Russia; sorokin@oncobox.com (M.S.); zolotovskaia.ma@mipt.ru (M.Z.); koroleva@oncobox.com (E.K.); krem-kristina@mail.ru (K.K.); buzdin@oncobox.com (A.B.); borisov@oncobox.com (N.B.); 2 Group of Experimental Biotherapy and Diagnostic, Institute for Regenerative Medicine, I.M. Sechenov First Moscow State Medical University, 119991 Moscow, Russia; margaret.kamynina@gmail.com (M.K.); dr.gudkov@gmail.com (A.G.); 3 OmicsWay Corporation, Walnut, CA 91789, USA; 4 Shemyakin-Ovchinnikov Institute of Bioorganic Chemistry, Russian Academy of Sciences, 117997 Moscow, Russia; 5 Oncobox Ltd., 121205 Moscow, Russia

**Keywords:** zinc, manganese, transitional elements, microelements, cancer, tumorigenesis, molecular pathways, cell growth signaling

## Abstract

Metal ion homeostasis is fundamental for life. Specifically, transition metals iron, manganese and zinc play a pivotal role in mitochondrial metabolism and energy generation, anti-oxidation defense, transcriptional regulation and the immune response. The misregulation of expression or mutations in ion carriers and the corresponding changes in Mn^2+^ and Zn^2+^ levels suggest that these ions play a pivotal role in cancer progression. Moreover, coordinated changes in Mn^2+^ and Zn^2+^ ion carriers have been detected, suggesting that particular mechanisms influenced by both ions might be required for the growth of cancer cells, metastasis and immune evasion. Here, we present a review of zinc and manganese pathophysiology suggesting that these ions might cooperatively regulate cancerogenesis. Zn and Mn effects converge on mitochondria-induced apoptosis, transcriptional regulation and the cGAS-STING signaling pathway, mediating the immune response. Both Zn and Mn influence cancer progression and impact treatment efficacy in animal models and clinical trials. We predict that novel strategies targeting the regulation of both Zn and Mn in cancer will complement current therapeutic strategies.

## 1. Introduction

Transitional metals, including iron, manganese and zinc, are widely employed in numerous biochemical reactions. According to the periodic table, Mn is located nearby Fe, with a similar size of atoms and electron configuration of outer orbitals (Mn: 127 pm, 3d5 4s2 and Fe: 128 pm, 3d6 4s2), thus suggesting similar, but not identical, chemical properties. In the biologically relevant 3+ ionization form, ionic radii and electron configurations are also similar: Mn^3+^: 72 pm 3d4 and Fe^3+^: 69 pM, 3d5. In contrast, Zn: 134 pm, 3d10 4s2 has the highest ionization energy among the transitional metals of the same period, and Zn^2+^, with the electronic configuration 3d10, is a predominant ion in biological systems. Nature exploits the low oxidation energy of transition metals to facilitate biochemical reactions that involve electron transfer or the reduction of molecules in processes such as the deactivation of reactive oxygen species (ROS) and mitochondrial energy production. Proteins interact with transitional metal by so-called coordination, in which acidic amino acids form non-covalent interactions with the ions of transition metals, thereby creating stable structures such as heme in hemoglobin and zinc fingers in zinc finger transcription factors. In general, because Fe^3+^ and Mn^3+^ are much more electrophilic than Zn^2+^, the former (Fe^3+^ and Mn^3+^) are readily used as electron acceptors, whereas Zn^2+^ is predominantly used as a coordination metal and rarely as a cofactor. Fe^3+^ is abundant in Earth, and, perhaps consequently, many more enzymes in humans use Fe^3+^ as a coordination metal; however, a few enzymes specifically need Mn^3+^ [1].

To function properly, cells need a particular “just right” combination of trace elements [2]. An excess of Mn^2+^ leads to toxicity due to accumulation in mitochondria ([3] and reference therein), and this is associated with the inhibition of mitochondrial enzymes [4,5] and the overproduction of H_2_O_2_ by mitochondrial superoxide dismutase [6]. Similarly to Mn^2+^, Zn^2+^ accumulates in mitochondria [7,8], intracellular vesicles [9], the endoplasmic reticulum and the Golgi [10,11]. Accordingly, exposure to high concentrations of Zn^2+^ overloads its intracellular depo and induces mitochondrial dysfunction and apoptosis [12,13,14]. In contrast, the exposure of normal cells to low levels of Zn^2+^ may have anti-oxidant and anti-apoptotic effects [15,16,17,18].

Low concentrations of Mn and Zn are necessary for normal cellular functions and are needed for proliferation [15], the inhibition [17] or induction of cell death [13], transcriptional regulation [19], ROS homeostasis [6,20,21] and keratinocyte differentiation [22], among others. To maintain and regulate Mn and Zn concentrations, multiple mechanisms are in place, including ion exchangers [23,24]; metallothioneins and glutathione buffering systems [25]; and the concentration of ions into vesicles, which can be transported out of cells [9,26,27].

Alterations in Mn and Zn homeostasis are associated with pathological conditions, such as cardiovascular diseases [28,29], neurodegenerative disorders [30] and autism spectrum disorders [31,32]. Recently, it became evident that alterations in Zn and Mn might be a factor that impacts cancerogenesis, e.g., in prostate cancer [33,34], colorectal cancer [35,36], lung cancers [37] and glioblastoma [38], among others [39]. Mutations and the altered expression of ion carriers that regulate Zn and Mn homeostasis are hallmarks of many cancers [40,41,42]. Intriguingly, cancers often exhibit coordinated changes in Zn^2+^ and Mn^2+^ ion carriers [40,43], although the way in which this modulates ion homeostasis or promotes cancer growth is yet to be investigated. In addition, the modulations of Mn and Zn influence the effectiveness of cancer therapeutics, and several clinical trials are currently underway [13,44,45,46] (clinical trial.gov NCT03991559, NCT04488783). Here, we discuss the mechanisms of the Zn- and Mn-mediated interactions that influence cancer metabolism related to new diagnostics and therapeutic applications [47,48,49].

## 2. Regulation of Zn/Mn Homeostasis

As discussed in the Introduction, alterations in Mn and Zn concentrations influence cell viability. Now, we want to discuss in more detail how Mn- and Zn-based regulation is built. To address this question, we need to analyze a few effects related to Mn and Zn homeostasis. First—what is the intracellular distribution of Mn and Zn, what is the range of the free and protein-bound concentrations of these ions in cells, and how are these concentrations regulated? Second—what is known about the biophysical mechanisms of Mn- and Zn-regulated biological activities inside cells, such as the affinities of proteins to Mn or Zn and reaction constants. Finally, what is known about the influences of Mn and Zn on the biological processes related to cancerogenesis?

### 2.1. Zn, Mn and ROS Detoxification Reactions

**Figure 1:**
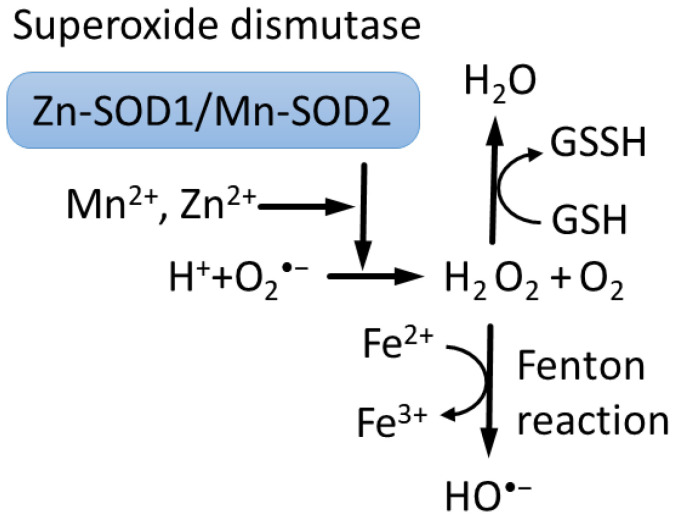
Superoxide dismutases (SODs) use ions of Cu^2+^ or Zn^2+^ and Fe^3+^ or Mn^2+^ to catalyze reduction of superoxide O_2_^•−^ to hydrogen peroxide H_2_O_2_ [6,52]. In turn, Fenton reaction converts hydrogen peroxide to hydroxyl •OH radical and hydroxide OH^−^ ions [53]. Moreover, hydrogen peroxide is converted into water by reduced glutathione (GSH), peroxiredoxins and catalase.

Importantly, Zn^2+^ and Mn^2+^ can catalyze the reduction of superoxide to H_2_O_2_ by superoxide dismutase, Cu/Zn-SOD1 in the cytoplasm and Mn-SOD2 in mitochondria [50], representing a major route of detoxification in cells (Figure 1) [2,6,51].

One important difference between Mn^2+^ and Fe^2+^ is that Fe^2+^ catalyzes the Fenton reaction, producing free radical HO^•^ [54,55], whereas Mn^2+^ does not. Thus, Mn^2+^ competition with Fe^2+^ can provide protection, in part, from oxidation-induced degradation [56]. However, mitochondria contain about 16µm of free Fe^2+^, which can participate in the Fenton reaction, promoting the toxicity of Mn^2+^-driven H_2_O_2_ overproduction in mitochondria, although this mechanism is debated [6,53,57,58]. It was recently discovered that the concentration of low-molecular-weight complexes of Mn^2+^ with orthophosphates or peptides is a dominant factor that predicts survival and that double-strand breaks repair efficiency after gamma irradiation across bacteria, fungi, archaea and human cells [59].

### 2.2. Cellular Distribution of Mn and Toxicity

A number of methods for the measurement of intracellular Mn concentrations have been reported in the literature. Interestingly, the addition of Mn to media leads to an increase in Mn concentration in the cell, in some reports way above concentrations in the media, suggesting active transport inside cells [6,60,61,62,63]. Using inductively coupled plasma–mass spectrometry, it was shown that the intracellular total Mn concentration in unexposed prostate cancer cells is about 1 μM and increases upon incubation with 1 mM of Mn for 48 h in PC3 (38.2 ± 14.3 μM), LNCaP (34.6 ± 0.7 μM) and DU145 cells (12.2 ± 0.4 μM) [60]. According to measurements using energy-dispersive X-ray fluorescence [61], the total Mn concentration in chick microglia was 45 μM in the presence of 0.4 μM of Mn and increased up to 100 μM with the addition of 2 μM of Mn. Measurements of Mn in blood cells using graphite-furnace atomic-absorption spectrophotometry with Zeeinan background correction revealed an Mn concentration equal to 0.3 μM in erythrocytes, 0.006 μM in polymorphonuclear and mononuclear leukocytes, and 0.016 μM in plasma [64].

It was shown that, upon exposure, Mn^2+^ accumulates mostly in mitochondria [3]; however, Mn^2+^ also binds DNA with Kd = 33 μm [65], and there is also accumulation in the nuclei, mostly in the heterochromatin [66]. Consistent with the mitochondria accumulation of Mn^2+^ upon exposure, isolated mitochondria are capable of sucking off the vast majority of exogenously added Mn^2+^ from media [3].

Currently, there are no indicators that would allow measurements of free Mn in living cells beyond the targeted probe [67]. Several Mn-specific molecules have been identified based on their properties to transfer Mn in or out of cells, thereby allowing the measurement of Mn release in media after the pre-loading of cells; however, it is difficult to interpret whether they are mitochondrial or nuclear Mn^2+^ or free cytoplasmic Mn^2+^ [68]. Because Mn^2+^ accumulates in mitochondria upon exposure [3], it is possible to speculate that the free Mn^2+^ concentration in uninduced cells is very low. It is not clear if Mn^2+^ can be released from mitochondria or other Mn^2+^ depos as it happens with Zn^2+^ in response to oxidative stress [9].

Cells typically tolerate up to 10 μm of Mn, and the addition of 50 μm or more for 24 h is toxic [6,60,62]. Notably, the addition of Mn to human neuroblastoma cells was found to be a dominant factor driving H_2_O_2_ production by mitochondrial Mn-SOD2 in the range of an extracellular MnCl_2_ concentration of 1–100 μM [6]. Upon the addition of extracellular Mn, the fraction of cellular Mn in total protein mass increased over the range of 6.4–50 × 10^−6^, which, according to the authors’ estimation, corresponds to normal physiological (6.4–36 × 10^−6^) and pathological (50 × 10^−6^) ranges. The addition of as little as 1 μM of Mn increased the mitochondrial oxygen consumption rate, H_2_O_2_ production and SOD2 activity. It was shown that the overexpression of Mn-SOD2 suppresses breast cancer growth in vitro and in xenograft models [69] and that the mimetics of Mn-SOD2 show anti-cancer activity [70], suggesting that Mn, in the contest of SOD activation, has anti-cancer effects. In contrast, recent research has demonstrated an increase in Mn-SOD2 in triple-negative breast cancer, leading to increased stemness and invasiveness of breast cancer cells and M2 macrophage invasion [71].

**Figure 2:**
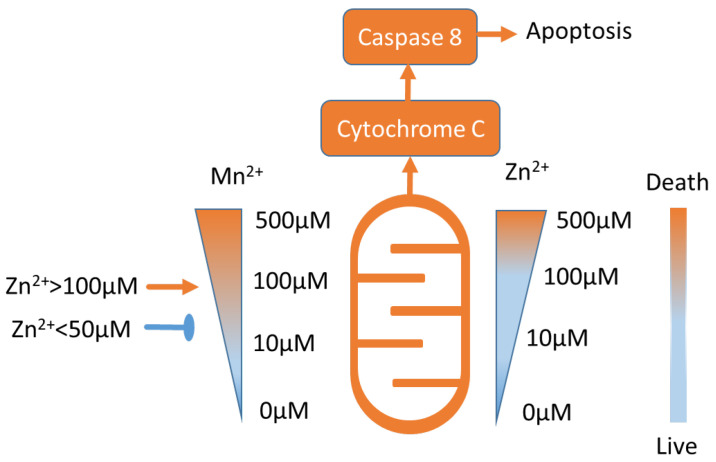
Approximate Mn^2 +^ and Zn^2+^ toxicity ranges (see text for details). Low concentrations of Mn^2 +^ and Zn^2+^ are not toxic. Notably, Zn^2+^ in concentrations lower than 50 μM inhibits Mn^2+^-induced toxicity, and Zn^2+^ in concentrations higher than 100 μM potentiates Mn^2+^ toxicity [73].

Apparently, a high concentration of Mn^2+^ (400 μM, 24 h) induces cytochrome C release from mitochondria and caspase-8-mediated apoptosis in B cells [2,72]. It was demonstrated that 10–50 μM of Zn prevents Mn-induced cell death, whereas a higher 100 μM concentration of Zn potentiates human Burkitt lymphoma B cell death (Figure 2) [73]. Similar data were obtained in murine photoreceptor cells [74], and the effect of Zn on cell viability is discussed in the following sections.

### 2.3. Regulation of Zn Homeostasis and Toxicity

In contrast to Mn, it is possible to measure free Zn concentration in cellular organelles using several indicators [11,75,76,77,78], and it is generally low, that is, in the order of 5–10 pM in PC12 cells [75] and 400 pM in pancreatic beta cells [76]. Most of the Zn in cells is bound by proteins, and it was estimated that approximately 3000 different proteins in cells bind Zn [79,80,81]. Among these, metallothionein serves as a major Zn^2+^ buffer, and several other proteins carry the same functions [25,82,83].

Gel filtration chromatography revealed three pools of Zn^2+^-bound molecules of different molecular weights: metallothionein, other proteins and reduced glutathione (GSH) [84,85]. A comparison of Zn^2+^ affinities for different pools of proteins revealed that apo-metallothionein was able to compete for 13% of Zn^2+^ bound to proteins, GSH competed for 10% of Zn^2+^ and synthetic chelators competed for 32–38% of Zn^2+^. Thus, the affinity of metallothionein to Zn^2+^ is relatively low in comparison to other Zn proteins; however, due to its abundance, it binds about one-third of cellular Zn^2+^. Thus, the Zn^2+^ available for protein binding in cells exists in the metallothionein-bound form. Accordingly, Zn depletion inhibits the activity of transcription factors, and supplementation with free Zn^2+^ or Zn^2+^ in the complex with metallothionein restores it [86,87].

Another one-third of cellular Zn is bound by other non-metallothionein proteins, and the rest of Zn is in the complex with glutathione [84,85,88,89]. It was also shown that the addition of GSH increases the speed of Zn^2+^ binding to proteins other than metallothionein [85,88].

Zn indicators can measure the intracellular distribution of Zn^2+^ [10], and it was revealed that, upon moderate exposure, Zn^2+^ is transferred by TRPM7 (transient receptor potential cation channel subfamily M member 7) into specific vesicles that release Zn^2+^ in response to oxidative stress [9] or in response to TRPM7 agonists, promoting melanoma cell death by inhibiting autophagy [13,90].

Upon exposure to high Zn^2+^ concentrations, cells efflux Zn^2+^ by lysosomal exocytosis [26] and accumulate Zn in mitochondria [7,8], where cell death is then induced [12,14]. The addition of Zn^2+^ to rat primary astrocytes and glioma cells induced GSH depletion, ROS and lactate dehydrogenase induction, mitochondria membrane depolarization and apoptosis [89]. The addition of Zn^2+^ or metallothionein as a Zn^2+^ or Cd^2+^ carrier inhibits membrane potential, ATP production and oxygen consumption by mitochondria [14,91,92,93,94].

### 2.4. Zn and Mn Transport

**Figure 3:**
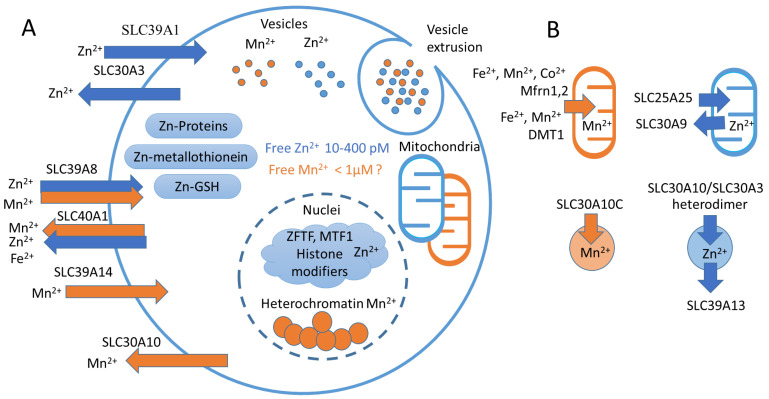
Mn^2+^ and Zn^2+^ homeostasis. (**A**) Representative scheme of Mn^2+^ and Zn^2+^ transport and cellular compartments. Blue arrows represent Zn^2+^-transporting carriers (SLC39A1, SLC30A3), orange arrows represent Mn^2+^-transporting carriers (SLC39A14, SLC30A10). Double arrows represent proteins that transfer both Mn^2+^ and Zn^2+^ (SLC39A8, SLC40A1). In the cell, Zn^2+^ is distributed between metallothioneins, other proteins and GSH [84,85]. In the nuclei, Zn^2+^ binds MTF1 [25] and serves as a coordination metal for the majority of histone-modifying enzymes [112,113] and zinc finger transcription factors (ZFTFs) [114]. In turn, Mn^2+^ accumulates in heterochromatin [66]. Both Mn^2+^ and Zn^2+^ can accumulate in mitochondria [3,8], as well as in the Golgi apparatus and endoplasmic reticulum [10,11,115,116] (not shown). Both Mn^2+^ and Zn^2+^ can be sequestered in specific vesicles that can be released from the cell [9,26,27]. (**B**) Ion carriers transfer Mn^2+^ in mitochondria using Mfrn1 [117] and DMT1 [118], and in cellular vesicles by SLC30A10 [108]. In turn, Zn^2+^ is accumulated in mitochondria by SLC25A25 and exerted by SLC30A9 [8,23]. In vesicles, Zn^2+^ is accumulated by SLC30A3-SLC30A10 heterodimer [107] and exerted by SLC39A13 [119].

The transport and distribution of zinc and manganese within cells are regulated by specialized transport proteins (Table 1 and Figure 3). They are conditionally divided into families of Zn importers ZIP/SLC39 and Zn exporters ZNT/SLC30 [11,43].

First of all, known Zn exporters use the proton gradient to transfer Zn^2+^ [105]; in contrast, the active transport of Mn^2+^ mediated by SLC30A10 is powered by the import of Ca^2+^ along the electrochemical gradient in exchange to Mn^2+^ across the electrochemical gradient [96]. Moreover, SLC30A10 is capable of transferring Mn^2+^ but not Zn^2+^ [96,106]. In contrast, SLC30A10, as a SLC30A3-SLC30A10 heterodimer, can transport Zn^2+^ and Mn^2+^ into endosomes and activate EGFR/MEK/ERK1,2 transduction, which were found to be reduced by the Zn^2+^ chelator TPEN [107]. In contrast to data from Levi [96], Zhao demonstrated that SLC30A10 overexpression influences Zn^2+^ transport [107].

After Mn^2+^ overexposure, WIF-B human/rat hybrid hepatocytes uptake Mn^2+^ from the cytoplasm by SLC30A10 into vesicles that fuse with the apical cell membrane and release their content in media [108]. Recent data suggest that, similarly to Zn^2+^ [26], Mn^2+^ is released from cancer cells by extracellular vesicles [27].

While SLC30A10 transports Ca^2+^ and Mn^2+^ in opposite directions, ATP2C1 transports Ca^2+^ or Mn^2+^ in the Golgi, preventing Mn^2+^-induced neurotoxicity [104]. The way in which Ca^2+^ entry in cells mediated by SLC30A10 influences Zn-regulated cellular physiology has not been investigated to date. Noticeably, Ca^2+^ entry in monocytes generates free cytoplasmic Zn^2+^ originating in the nuclear and perinuclear endoplasmic reticulum regions [109] or in the mitochondria in neurons [7].

The induction of Zn^2+^ release seems to be required for and precedes ROS generation in mitochondria in response to hypoxia [21,110]. It was reported that Hif1a activation is required for the induction of SLC30A10 expression upon Mn^2+^ exposure [111]. Hif1a is induced by hypoxia and ROS, and it would be interesting to investigate if SLC30A10 is induced in cancers by similar mechanisms [40].

### 2.5. Zn and Mn Transporters and Cancerogenesis

The major insights into the functions of zinc transporters in cancerogenesis come from cancer genetics studies [39,120]. In short, the majority of these studies demonstrated reduced Zn levels in different cancers, as well as the corresponding downregulation of importers and the upregulation of exporters [120].

In prostate cancer, zinc deficiency occurs due to the downregulation of zinc transporters [121], and the treatment of prostate cancer cells with physiological concentrations of Zn induces apoptosis [122]. Specifically, the overexpression of Ras-responsive element-binding protein 1 (RREB1) downstream of the Ras-Raf-MEK-ERK signaling pathway represses SLC39A1 (ZIP1) expression leading to a reduced Zn level [123,124]. Preclinical models support the application of Zn^2+^ ionophore clioquinol in combination with a dopamine agonist for prostate cancer treatment [41,125]. In contrast, RREB1/SLC39A3(ZIP3)/Zn were all found to be downregulated in pancreatic adenocarcinoma [126], and a low Zn level was associated with hyperproliferation in vitro. In ovarian cancer, SLC39A13 (ZIP13) and ZIP5, ZIP10, ZIP12 and ZIP14 overexpression were found to be associated with poor prognosis, and SLC39A13 knockout demonstrated suppression of the malignant phenotype in vitro and in vivo and a higher vesicular zinc level in knockout cells [127]. The expression of 10 members of the SLC30 family was measured in cervical carcinoma and revealed a gradual induction of the Mn^2+^ effluxer SLC30A10 with an increase in cancer stage; complete depletion of the Zn^2+^ vesicular transporter SLC30A8; and the induction of Zn^2+^ exporters SLC30A1, SLC30A6 and SLC30A7 [40].

In turn, due to the superoxide radical scavenger properties of Mn^2+^ [59], high levels of Mn in cancers are associated with poor survival and low radiosensitivity of tumors, such as for melanoma and glioblastoma, in comparison to classical seminoma, breast cancer and prostate cancer [38]. Mn distribution across tissue sections was measured using mass spectrometry. No such correlation was observed for Zn, Cu and Fe in that study. The scanning of tissue sections from the Lewis lung carcinoma metastasis mice model using a similar approach revealed Mn accumulation in a few foci in the primary tumor or in the tissues of untreated animals, whereas the distribution of Zn was uniform [27]. Intriguingly, a higher Mn concentration was detected in organs from the tumor-bearing mice. In addition, sub-toxic 5 µM levels of Mn^2+^ promote cell migration, and exosomal Mn^2+^ exertion was detected [27]. Whether these high Mn regions in the organs of tumor-bearing mice represent metastasis sites or only Mn accumulation requires further investigation.

In this regard, it was demonstrated that Mn^2+^ is the main substrate for SLC39A14 in vivo using mice studies [128] and in humans [100,129], and it is associated with Mn accumulation in blood and most other organs while depleting in the liver. Accordingly, the decreased expression of SLC39A14 was associated with aggressiveness and the relapse of prostate cancer [130], and alternative splicing of SLC39A14 was associated with colorectal cancer [131,132]. However, some studies have found an increase in both Zn and Mn in colorectal cancer tissue, while others observed only a slight difference in Zn levels in males [133,134].

### 2.6. Transcriptional Regulation by Zn and Mn

Both Zn^2+^ and Mn^2+^ regulate transcription. In neuronal cells, Mn^2+^ induces apoptosis in PC12 cells facilitated by caspase 3 transcriptional activation triggered by the phosphorylation of zinc finger transcription factor SP-1 [135]. In addition, in PC12 cells, Mn^2+^ potentiates histone deacetylase (HDAC) and represses histone acetyltransferase (HAT) activities, leading to the inhibition of the acetylation of core histones [19], which is consistent with the localization of Mn^2+^ in heterochromatin compartments [66]. The inhibition of HDAC activity was found to attenuate cell death, and the inhibition of HAT was found to potentiate Mn^2+^-induced cell death, suggesting the role of histone acetylation in Mn-induced dopaminergic neurotoxicity [19].

Zinc can regulate transcription because it is a component of many if not all chromatin remodelers, including HDAC, HAT [112,113] and histone demethylases [136,137]. In contrast to Mn in PC12 cells, both Zn^2+^ and zinc transporter ZIP10 activate HAT in keratinocytes, promoting differentiation and the expression of metallothionein genes [22]. Mechanistically, Zn directly regulates the metal-responsive transcription factor 1 (MTF1)-mediated induction of metallothionein genes, thereby generating more Zn^2+^ storage in response to the increase in Zn^2+^ in the environment [25].

A recent paper reports that HDAC8 activity can be regulated by competition between different ions, specifically, Zn^2+^ and Fe^2+^ [113]. The HDAC8 active site has similar architecture to the arginase Mn^2+^ site, in which a single catalytic Zn^2+^ ion is coordinated by two aspartate residues and a histidine [113,138]. HDAC8 exhibits 10^6^ higher affinity to Zn^2+^ than to Fe^2+^ (Zn^2+^ (Kd = 9 pM); Fe^2+^ (Kd = 1.1 μM)), compensating for the higher Fe^2+^ concentrations in cells and higher catalytic activity of HDAC8 in the presence of Fe^2+^. In addition, HDAC8 can also bind Mn^2+^ and Cu^2+^, but a comparison of affinities or catalytic activities was not performed [113,138].

Consistent with the roles of Zn [22] and DNA methylation in keratinocyte differentiation [139,140], DNA methyltransferase 1 activity was found to be induced by the depletion of dermis zinc transporter SLC39A13 (ZIP13), and the effect was reversed by Zn supplementation [141].

Consistent with the high affinity of most of the proteins to Zn, the affinity of the third zinc finger of SP-1 to Zn was characterized by Kd = 6 × 10^−10^, much higher than that for metallothionein [114]. The activity of the Zn-SP-1 transcription factor was evaluated when cellular zinc was depleted using a series of ligands, including apo-metallothionein, glutathione, EDTA, EGTA and TPEN [84]. Out of these, only cell-permeable TPEN at 30 µm was able to inhibit SP-1 binding to DNA in nuclear extracts after 24 h of treatment and completely inhibit DNA binding in vitro. In contrast, Zn inhibited NF-κB activity, which could be attenuated by an increased metallothionein level [142]. At the same time, the Zn^2+^-mediated inhibition of Nf-κB has a profound effect on cancer progression, inhibiting proliferation and inflammation [143,144,145].

### 2.7. Mn- and Zn-Mediated Signal Transduction Pathways

In an attempt to reconstitute the Zn-mediated signaling network, the levels of gene expression in response to Zn^2+^ were measured in human intestinal Caco-2 cells with depleted MTF1 [25]. Interestingly, the majority of Zn^2+^-regulated genes augmented their response, and only metallothioneins and zinc-effluxing Znt1 were less sensitive to Zn^2+^ in the absence of MTF1. This suggests that effective Zn^2+^ levels in the cells became higher due to the diminished buffering capacity of metallothioneins and the efficiency of Zn^2+^ transport out of the cells. This places MTF1 on the top of the Zn-mediated signaling pathway followed by metallothioneins and other Zn-regulated genes. A mathematical model that describes Zn^2+^ homeostasis in cells has been formulated [85].

Zn is known to regulate cancer-related signaling pathways. External Zn^2+^ activates ERK signaling cascades and Ras [146]. Another investigation demonstrated that serum Zn^2+^ represses proapoptotic p38 and JNK signaling, which are activated by the mutant hRas *G12V* [147]. Nf1 is a classical Ras repressor [148,149], and Zn^2+^ coordination closes Nf1 domains to repress wild-type Ras-GTPase activity in vitro [150].

There are few publications suggesting that Mn^2+^ induces apoptosis or senescence by p53-dependent mechanisms [151,152,153]. Interestingly, in neuronal cells, Mn^2+^-induced toxicity was accompanied by increased p53 and mitochondrial p53 localization, while an increase in ROS and mitochondrial H_2_O_2_ production was attenuated by p53 inhibitors [151]. Similarly, in colorectal cancer cells, Mn-SOD2 overexpression induces p53-dependent senescence [153]. In addition, Mn^2+^-induced apoptosis was repressed by the DNp73 isoform of the p53 family member p73 [152,154].

The way in which Mn^2+^ regulates p53 activity is not known. One candidate for such regulation is the p53 activator protein phosphatase PP2Cα [155], which is characterized by the apparent Mn^2+^ Michaelis constant, Kmetal = 3.3 mM, far above typical total concentrations in cells, and the substitution of Mg^2+^ with Mn^2+^ was found to decrease the activity of the enzyme by a factor of 30 [156]. In the contest of p53-dependent apoptosis, it would be interesting to examine if p53 phosphorylation occurs downstream of PP2C activity and the role of the Mg/Mn ratio in the cells.

**Figure 4:**
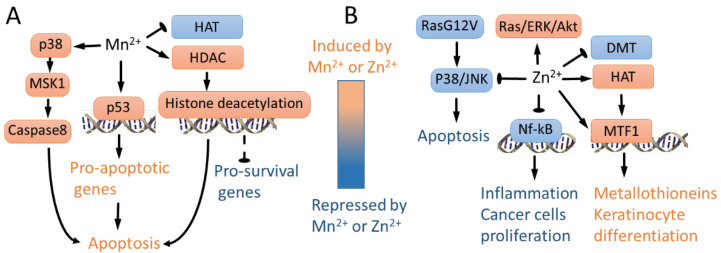
Mn^2+^- and Zn^2+^-regulated signaling pathways. (**A**) Mn^2+^ represses global histone acetylation by augmenting HDAC and repressing HAT activity, leading to apoptosis presumably due to repression of key pro-survival genes [19]. In addition, Mn^2+^ activates p53 [151,152,153] and p38/MSK1 signaling [72], leading to apoptosis. (**B**) In wild-type Ras cells, Zn^2+^ activates ERK/AKT signaling [146]. In contrast, in mutant G12V RAS cells, Zn^2+^ inhibits p38 and JNK, repressing apoptosis [147]. Zn^2+^ inhibits Nf-kB, leading to lower proliferation of cancer cells and inhibiting inflammation [144,145,157]. In contrast to Mn^2+^, Zn^2+^ activates HAT, leading to metallothionein induction and keratinocyte differentiation-specific gene expression [22]. Consistently, Zn^2+^ represses DNA methyltransferase activity in immortalized mouse fibroblasts [141]. Zn directly regulates MTF1-mediated induction of metallothionein genes [25], leading to expression of keratinocyte differentiation genes [22].

Besides the p53 activation discussed above, Mn^2+^ induces apoptosis by caspase-8 activation downstream of p38-MSK1 signaling in human B cells [72]. To conclude, Mn^2+^ and Zn^2+^ affect several signaling pathways (p38 and the regulation of histone acetylation) in opposite directions; however, others affect cells in a similar way, including the induction of 53 by Mn^2+^, the inhibition of Nf-κB-induced proliferation [144,145,157] and the induction of keratinocyte differentiation by Zn^2+^ ions (Figure 4).

### 2.8. Differential Effects of Mn and Zn in Normal and Cancer Cells

It was found that zinc sulfate was not toxic to normal cells at 100 µm or below after four days of cultivation, but it significantly decreased the viability of myelogenous leukemia K562 cancer cells at 40 µm and above [158]. It was demonstrated that zinc sulfate protects normal lymphocytes from H_2_O_2_-induced DNA damage and augments H_2_O_2_-induced DNA damage in K562 cancer cells [158]. The same effect was demonstrated in a recent study of acute myeloid leukemia cells [159]. Similarly, the application of temozolomide and ZnCl_2_ at 100 µm enhanced the treatment efficiency of cells and the xenograft model of glioblastoma and did not affect normal human astrocytes [46]. Clinical trials of Zn supplementation in glioblastoma in parallel to temozolomide treatment are currently ongoing (cliniclaltrilas.gov NCT04488783). A manganese compound Adpa-Mn was found to preferentially induce autophagy and the death of glioblastoma and other cancer cell lines but not astrocytes or non-malignant cells [160,161].

## 3. Effect of Zn and Mn on cGAS-STING Pathway, Immune Response and Cancerogenesis

**Figure 5:**
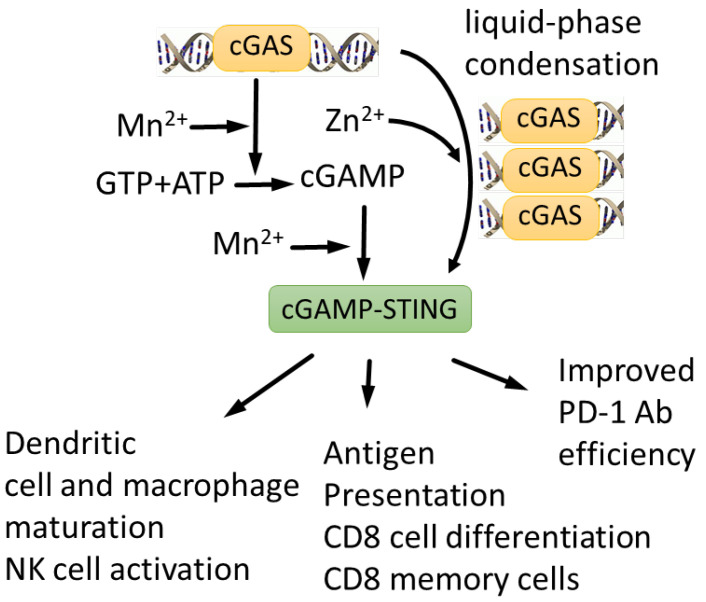
cGAS-STING signaling pathway is activated by both Mn^2+^ and Zn^2+^. Mn^2+^ is required for efficient DNA sensing, cGAMP synthesis and cGAMP-STING complex formation [63], whereas Zn^2+^ is required for cGAMP folding and liquid-phase separation of cGAMP-DNA complexes [172,173,174]. Mn^2+^ potentiates cGAS-STING anti-tumor response, stimulating dendritic cell and macrophage maturation, NK cell activation, antigen presentation and CD8 cell differentiation [44]. As a result, Mn^2+^ enhances efficiency of therapeutic antibodies [170].

In addition to coordinately regulating ROS response, transcription and metabolism, Zn^2+^ and Mn^2+^ together regulate a component of the innate immune system, namely, the cGAS-STING (cyclic GMP-AMP synthase—Stimulator of Interferon Genes) pathway, which is activated by cytoplasmic double-stranded DNA caused by viral infection [162] (Figure 5). The cGAS-STING pathway is also involved in the p21-mediated DNA damage response [163] and chromatin stabilization during mitosis upon genotoxic drug treatment [164,165]. cGAS-STING activation requires Mn^2+^ release from intracellular organelles, presumably mitochondria [63]. Mn^2+^ binds to cyclic GMP-AMP synthase (cGAS) and enhances its sensitivity to double-stranded DNA and the production of the secondary messenger cyclic GMP-AMP (cGAMP), leading to NFkb activation and antiviral response [63,166,167,168,169]. It was recently shown that Mn^2+^ is involved in the anti-tumor immune response activated by the cGAS-STING pathway [44]. Mn^2+^ activated both innate and adaptive arms of the immune system, repressed metastasis and potentiated immune checkpoint therapy in mice. A dose-escalating phase 1 clinical trial to estimate the safety and preliminary efficacy of Mn^2+^-primed anti-PD-1 treatment and chemotherapy is currently ongoing (clinical trial.gov NCT03991559) [44].

In addition, Mn^2+^ potentiates the effect of the TGF-β/PD-L1 bispecific antibody YM101 against several in vivo cancer models by activating the STING pathway and promoting the maturation of mouse and human dendritic cells, shifting the tumor microenvironment toward the inflamed phenotype. This enhances the antigen presentation, infiltration and function of T-lymphocytes [170]. The therapeutic activity of YM101 and Mn^2+^ administration was demonstrated using mice models of hepatocellular carcinoma (H22), melanoma (B16), colon (CT26) cancer and breast (EMT-6) cancers [170]. The application of self-assembled cyclic dinucleotide STING agonists and Mn^2+^ nanoparticles induced anti-tumor immunity and a remarkable therapeutic effect in multiple tumor models [171].

In turn, Zn^2+^ is also involved in cGAS-STING regulation. Zn^2+^ coordinates the cGAS ribbon, which is essential for the interferon response. Zn^2+^ coordination is also required for cGAS–DNA liquid-phase condensation and cGAMP production [172,173,174], whereas cGAS binding to DNA is augmented by the zinc finger protein ZCCHC3 [175].

To conclude, recent research supports an anti-cancer role of Zn and Mn via modulation of the inflammatory pathways and immune reactions [44,49,176].

## 4. Conclusions and Future Perspectives

Transition metal ions Mn^2+^ and Zn^2+^ have profound anti-cancer effects (Table 2). However, their biochemical properties and physiology are different, although with a certain degree of similarity with respect to regulated proteins and metabolic pathways. Taking into account that Mn^2+^ transport proteins also transport Zn^2+^ and other ions, and that a few Mn^2+^- or Zn^2+^-specific transporters exist, it is possible to speculate that there are common mechanisms that are regulated by and regulate these ions. Among the few mechanisms commonly influenced by both Mn and Zn are effects on mitochondrial function, including ROS detoxification and the induction of apoptosis, transcriptional regulation and chromatin dynamics, cGAS-STING-mediated apoptosis and the immune response (Table 3).

Many intriguing questions remain to be addressed in future studies. First, conflicting data regarding Zn and Mn concentrations in cancers prompt more detailed studies, in which levels of Zn and Mn in different types of tumor cells should be examined, such as cancer cells and different types of immune and stromal cells from the tumor microenvironment. To address the functions of Mn^2+^ in cell physiology, the field needs to develop intracellular Mn^2+^ probes to be able to monitor the Mn level during investigations in live cells, in a similar fashion to how it is performed for Zn^+2^. In future experiments, it would be interesting to examine in more detail how Mn^2+^- and Zn^2+^-mediated effects are interconnected. For example, cGAS-STING activation is affected by both Zn^2+^ and Mn^2+^ by different mechanisms, and an investigation of the relative impact of these on apoptosis and cancer progression would be interesting.

We suggest investigations of the combinatorial effects of Zn and Mn on cancers where individual ions have some effect [13,44,45,46,171]. Recently developed Zn–Mn-composed nanoparticles with potential anti-cancer effects might be useful for such studies [177,178,179,180].

Finally, details of such investigations will lead to pre-clinical research and, hopefully, to clinical trials examining the effect of combined Mn and Zn supplementation on the efficiency of anti-cancer drugs.

## Figures and Tables

**Table 1:** Representative Mn^2+^ and Zn^2+^ ion carrier genes.

Gene Name	Protein Name	Specificity	Type of Transport	Reference
*SLC30A10*	ZNT10	Mn^2+^/Ca^2+^ exchange, Zn^2+^ vesicular transport as SLC30A3 heterodimer	Exporter	[95,96]
*SLC30A3*	ZNT3	Zn^2+^	Exporter	[39,97,98,99]
*SLC39A14*	ZIP14	Divalent metal cations Mn^2+^, Zn^2+^, Fe^2+^	Importer (symport)	[97,100,101,102]
*SLC39A8*	ZIP8	Mn, Zn, Fe	Importer (symport)	[97,100,103]
*TP2C1*		Ca or Mn	Mitochondrial influx	[104]

**Table 2:** Pivotal findings of Mn^2+^ and Zn^2+^ roles in cancer physiology in vivo.

Main Finding	Reference
Certain cancers exhibit coordinated changes in Zn^2+^ and Mn^2+^ carriers	[40,43]
High levels of Mn in cancers are associated with poor survival and low radiosensitivity of tumors, such as for melanoma and glioblastoma	[38]
Preclinical models support application of Zn^2+^ ionophore clioquinol in combination with dopamine agonist for prostate cancer treatment	[41,125]
Mn^2+^ boosts innate and adaptive anti-cancer immune response and boosts PD-1 immunotherapy	[44]
Therapeutic activity of YM101 and Mn^2+^ was demonstrated using mice models of hepatocellular carcinoma, melanoma, colon cancer and breast cancers	[170]
Zn^2+^ enhances temozolomide efficiency in glioblastoma xenograft model	[46]

**Table 3:** Pivotal findings of Mn^2+^ and Zn^2+^ roles in cancer physiology in vitro.

Main Finding	References
Mn^2+^-SOD2 drives H_2_O_2_ production in mitochondria in a wide range of extracellular concentrations	[6]
Mn^2+^ at high concentrations induces mitochondrial cell death	[72,151]
Zn^2+^ at high concentrations induces mitochondrial cell death	[13,74]
Zn^2+^ at low concentrations inhibits Mn-induced mitochondrial cell death	[73]
Low-molecular-weight complexes of Mn^2+^ predict cell survival, and double-strand breaks repair efficiency after gamma irradiation	[59]
Zn^2+^ release is required for and precedes ROS generation in mitochondria in response to hypoxia	[21,110]
Mn^2+^ activates p38/MSK1-regulated apoptosis	[72]
Zn^2+^ inhibits p38 and JNK and represses apoptosis in mutant G12V RAS cells	[147]
Zn^2+^ activates RAS signaling cascade	[146]
Mn^2+^ induces apoptosis or senescence by p53-dependent mechanisms	[151,152,153]
Zn^2+^ represses NF-κB activity and sensitizes prostate cancer cells to cytotoxic agents	[144,145,157]
Mn^2+^ represses histone acetylation by repressing HAT activity and augmenting HDAC, leading to apoptosis	[19]
Zn^2+^ activates HAT and MTF1-mediated transcription, leading to metallothionein induction and keratinocyte differentiation	[22]
Mn^2+^ is indispensable for cGAS-STNG activation and host defense against DNA viruses	[63]
Zn^2+^ coordination is required for cGAS–DNA liquid-phase condensation and cGAMP production	[172,173,174]

## Data Availability

Not applicable.
